# Estrogen downregulates TAK1 expression in human fibroblast-like synoviocytes and in a rheumatoid arthritis model

**DOI:** 10.3892/etm.2022.11149

**Published:** 2022-01-17

**Authors:** Xi Li, Miao Li

Exp Ther Med 20:1764–1769, 2020; DOI: 10.3892/etm.2020.8848

Following the publication of the above article, the authors have realized that they included the incorrect data for the GAPDH western blot bands in Fig. 2B on p. 1766.

The authors have examined their original data, and have been able to identify the GAPDH bands that should have been included in this figure. The corrected version of Fig. 2 is shown opposite. Note that the revised control data shown for this figure do not affect the overall conclusions reported in the paper. All the authors agree with the publication of this corrigendum, and are grateful to the Editor of *Experimental and Therapeutic Medicine* for allowing them the opportunity to publish this. They also apologize to the readership for any inconvenience caused.

## Figures and Tables

**Figure 2:**
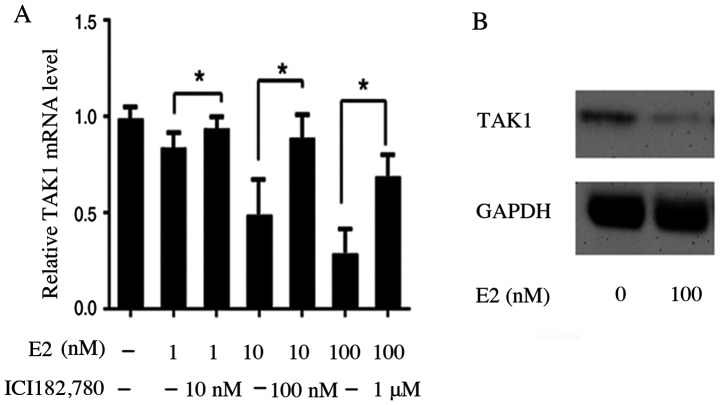
Effect of E2 on TAK1 mRNA and protein expression in FLS. (A) FLS were exposed to E2 with or without the ER antagonist ICI 182,780 for 24 h. TAK1 mRNA was measured by reverse transcription-quantitative PCR analysis. Data are presented as relative expression units, normalized to GAPDH. Values are the mean ± standard error of the mean from three independent experiments. ^*^P<0.05. (B) TAK1 and GAPDH levels were determined by western blot analysis of FLS that were incubated with or without E2 for 24 h. Data are presented as the mean ± standard error of the mean. TAK1, transforming growth factor β-activated kinase-1; FLS, fibroblast-like synoviocytes; E2, 17β-estradiol.

